# Integrated expression quantitative trait loci and Mendelian randomization analyses of the candidate genes and pathways identified for myocardial infarction

**DOI:** 10.3389/fmolb.2026.1693113

**Published:** 2026-03-11

**Authors:** Peichun He, Xiangwen Lv, Chuwen Fu, Zhen Qin, Siyuan Chen, Jinmin Zhao, Jian Xie

**Affiliations:** 1 School of Basic Medical Sciences, Guangxi Medical University, Nanning, Guangxi, China; 2 Department of Cardiology, The Second Affiliated Hospital of Guangxi Medical University, Nanning, Guangxi, China; 3 Department of Cardiology, The First Affiliated Hospital of Guangxi Medical University, Nanning, Guangxi, China; 4 Department of Orthopaedics Trauma and Hand Surgery, The First Affiliated Hospital of Guangxi Medical University, Nanning, Guangxi, China; 5 Collaborative Innovation Centre of Regenerative Medicine and Medical BioResource Development and Application Co-constructed by the Province and Ministry, Guangxi Medical University, Nanning, China

**Keywords:** biomarkers, candidate genes, expression quantitative trait loci, Mendelian randomization, myocardial infarction

## Abstract

**Background:**

Myocardial infarction (MI) is a myocardial necrosis event caused by an unstable ischemic state that reduces life expectancy primarily through cardiac functional impairment and cardiomyocyte death. The present study aims to investigate the genetic mechanisms underlying MI by integrating expression quantitative trait loci (eQTLs) and Mendelian randomization (MR) analyses.

**Methods:**

We comprehensively analyzed independent MI datasets from the Gene Expression Omnibus database. The relationships between MI and the differentially expressed genes were evaluated through differential expression, eQTL, and MR analyses. Additionally, GO and KEGG enrichment analyses were performed to clarify the functional pathways of the candidate genes, and gene set enrichment analysis (GSEA) was used to identify the genes associated with MI. An *in vitro* model of MI was established by subjecting AC16 cells to oxygen and glucose deprivation, and the gene expression levels were validated through reverse transcription quantitative polymerase chain reaction (RT-qPCR).

**Results:**

By comparing the results from the MR analysis and mRNA expression profiles, we identified 13 overlapping genes: *MRPL35*, *SNUPN*, *ADM*, *BCL6*, *BNIP3L*, *CMTM2*, *DGAT2*, *HSPA6*, *IER3*, *IFNGR1*, *PLAUR*, *SERPINB8*, and *VNN2*. The GO and KEGG enrichment analyses revealed that these genes participate in essential biological processes, including mitochondrial apoptotic and mitochondrial organization regulatory pathways. GSEA demonstrated that the candidate genes were enriched in the NOD-like signaling pathways; immunological responses; and lysosomal, ribosomal, and metabolic pathways related to MI. Furthermore, the gene expression levels were verified through RT-qPCR.

**Conclusion:**

This study highlights the potential of specific molecular pathways for targeted treatment of MI. Our work also warrants additional research efforts to elucidate the genetic mechanisms of MI.

## Introduction

Myocardial infarction (MI) is a necrosis event of the cardiac musculature induced by unstable ischemic syndromes and has emerged as a severe public health concern because of its high morbidity, disability, and fatality rates (Anderson and Morrow, 2017). MI occurs primarily because of various factors like heredity, poor lifestyle, and chronic diseases that lead to coronary artery atherosclerosis, plaque rupture, and thrombus formation, causing acute and persistent ischemia as well as hypoxia of the coronary arteries to eventually trigger MI (Xue et al., 2022). The epidemiological traits of MI have changed substantially in recent years; the prevalence of MI continues to increase every year among individuals under the age of 45 years as younger patients are being affected more frequently (Krittanawong et al., 2023). Despite the success of thrombolytic therapy and standardized treatments like percutaneous coronary intervention and coronary artery bypass grafts to reduce the size of the infarcted area and increase the overall prognosis, MI remains the leading cause of heart failure and increased mortality following left ventricular remodeling (Jiang et al., 2022; Kologrivova et al., 2021). Hence, a deeper understanding of the molecular pathways underlying the development of MI is needed to achieve the intended preventive and therapeutic goals.

Expression quantitative trait loci (eQTLs) analysis can be used to estimate the associations between genetic variants like single-nucleotide polymorphisms (SNPs) and the RNA expression of genes, which link the variants to regulated target genes to understand the action modes of the genetic variants. It also provides an efficient method of exploring the candidate genes related to complex human traits or diseases as well as identifying opportunities for therapeutic interventions (Zhu et al., 2016). Mendelian randomization (MR) is an advanced data analysis technique that is widely used in epidemiological studies to infer the cause of an event. This technique uses genetic variants that are closely linked to exposure factors as instrumental variables to accurately assess the potential causal relationships between exposure and outcome. In recent years, MR has been increasingly used to predict the safety and efficacy of new and existing drugs targeting cardiovascular risk factors (Daghlas and Gill, 2023).

The present study integrates eQTL data with MR analysis to identify candidate genes and biological processes that offer fresh theoretical groundwork and potential treatment options for MI.

## Materials and methods

### Data sources and processing

The GSE66360 dataset (Muse et al., 2017), which primarily comprises gene expression data from gene chips (microarrays) derived from the circulating endothelial cells of 49 MI patients and 50 healthy controls, was downloaded from the Gene Expression Omnibus (GEO) database (https://www.ncbi.nlm.nih.gov/geo/). Perl (http://www.perl.org) was used to convert and annotate the GSE66360 dataset, and the probe names were translated to gene names.

### Identification and enrichment analysis of differentially expressed genes (DEGs)

The “limma” package in R was used to identify the DEGs, which were determined as candidates with |log_2_ (FC)| ≥ 0.585 and adjusted *p* < 0.05. Following analysis of the expression levels, the “ggplot2” and “heatmap” R packages were used to produce volcano maps and DEG heatmaps to screen and identify the expression levels of the DEGs associated with the MI and control groups.

### Genome-wide eQTL dataset

The eQTLs are genetic loci known to regulate gene expression. Using an eQTL as an instrumental variable allows observation of the phenotype changes induced by differences in the gene expression. eQTL analysis was used in this study to detect genetic expression variations by combining transcriptome and genotyping data from multiple cohorts. All the data examined in this study were obtained from publicly available sources, and the eQTL data were sourced from the eQTLGen collaboration (https://eqtlgen.org/). The “TwoSampleMR” R package was used to identify SNPs with strong associations (*p* < 5e-8) as the instrumental variables (Purcell et al., 2007). The linkage disequilibrium settings were adjusted such that r^2^ < 0.001 and clumping distance = 10,000 kb (Kuppa et al., 2023). Furthermore, SNPs with weak relationships or insufficient explanations for phenotypic variance were eliminated using the filter “F test value > 10” (Bowden et al., 2016).

### Determination of outcome data

The genome-wide association studies (GWAS) summary dataset and genetic association database were used to retrieve the outcome data (https://gwas.mrcieu.ac.uk/). We searched the GWAS database for MI, and the dataset with the ID “finn-b-19_MI” was chosen as the final data based on the sample size, ethnicity, sequencing depth, and data updating period. In brief, our GWAS samples include data from 200,641 European individuals, including 12,801 MI patients. The individually preserved blood samples subjected to DNA extraction and genotyping were obtained from the UK Biobank database. The genotype imputation was performed using the Axiom array IMPUTE4 (https://jmarchini.org/software/) (Feng et al., 2022). The GWAS part of this work was carried out using linear mixed models to prevent the removal of numerous related individuals from the UK Biobank database. All GWAS summary statistics used in this work are publicly available and accessible. Furthermore, the requisite ethical approvals were obtained during the original analyses.

### MR analysis

MR analysis was performed using the “TwoSampleMR” package in R software. The inverse variance-weighted (IVW) approach was used to examine the connections between MI and specific genes. Additional sensitivity analyses were performed using the MR-Egger, simple mode, weighted median, and weighted mode approaches (Dudbridge, 2021; Zhu et al., 2016). Finally, three criteria were used to identify the disease-linked genes as follows: initially, genes with an IVW *p*-value of less than 0.05 were chosen; the genes were further narrowed by the directional consistency of the odds ratio (OR) values (MR analysis results) across three different approaches; genes showing evidence of pleiotropy and having *p*-values less than 0.05 were excluded from the selection process.

Following this selection procedure, genes, the expression of which was either upregulated or downregulated, were identified through the intersection between the disease-related genes and DEGs. To determine the causal relationship between each intersecting gene and the illness, subsequent MR studies were performed on each gene separately. To assess the robustness and dependability of the findings, our investigations included leave-one-out sensitivity analyses, heterogeneity tests, and pleiotropy tests. The instrumental variant intercepts were used as the basis for the MR-Egger studies, and the weighted median technique allowed accurate causal estimation. MR-PRESSO was used to remove the outliers and account for horizontal pleiotropy. We also performed the leave-one-out sensitivity analysis to determine whether the results were affected by any SNPs. For the MR analysis, the “TwoSampleMR,” “MendelianRandomization,” “MR-PRESSO,” and “MR.raps” packages were used (Sun et al., 2024). To visually represent and support our findings, we created scatter plots, forest plots, and funnel plots for the analyses.

### Functional enrichment analyses of the candidate genes

The chromosome distribution circles for the hub genes were mapped using the “RCircos” package, whose source code can be downloaded and installed from the CRAN website (http://www.r-project.org) under the GPL license (≥2). The graphical functions were handled using basic R graphics (Zhang et al., 2013). Additionally, to investigate the biological functions and specific mechanisms of the MI-associated hub genes, we performed Gene Ontology (GO) and Kyoto Encyclopedia of Genes and Genomes (KEGG) pathway enrichment analyses using the clusterProfiler package, where a threshold of *p* < 0.05 was considered as significant enrichment. The “enrichplot” package was used to obtain various visualizations, such as histograms, bubble plots, and chord plots.

### Gene set enrichment analysis (GSEA)

The identified genes were arranged by phenotypic relatedness, and the distribution trend of the genes belonging to a preset gene set was evaluated by GSEA. Here, we used GSEA to determine the correlations between the identified genes and all other genes in the dataset. Once the correlations were computed in batches, all genes were categorized by correlation from high to low values, which resulted in a set of test genes that were more strongly correlated with the featured genes. Next, we clarified the changes in the signaling pathways among the candidate genes in the high- and low-expression groups. To elucidate the impacts of synergistic gene modifications within this set on the functional pathways, we clarified the signaling pathway changes in the candidate genes in the differential high- and low-expression groups. Simultaneously, a KEGG signaling pathway set was used as a predefined gene set. We retrieved “c2.cp.kegg.v7.5.1.symbols.gmt” from the Molecular Signatures Database (MSigDB) (https://www.gsea-msigdb.org/gsea/msigdb/) to assess the associated pathways and molecular processes after grouping the samples on the basis of the hub genes. The cutoff criteria included a false discovery rate (FDR) < 0.25 and *p*-value < 0.05.

### Cell culture and construction of the cellular model of MI

AC16 cells were obtained from a cell bank (Procell, China) and cultured in Dulbecco’s modified Eagle’s medium (DMEM, Gibco, MA, United States) supplemented with 1% penicillin/streptomycin and 10% fetal bovine serum at 37 °C under a 5% CO_2_ atmosphere. To simulate ischemia *in vitro*, the AC16 cells were divided into the control (Con) and oxygen–glucose deprivation (OGD) treatment groups. The OGD samples were cultured in a glucose-free medium under an anoxic atmosphere (95% N_2_ and 5% CO_2_) for 6 h.

### Reverse transcription quantitative polymerase chain reaction (RT-qPCR) analysis

TRIzol reagent (Invitrogen, CA, United States) was used to extract the RNA from each sample group. The RNA was then reverse transcribed into cDNA using the QuantiTect reverse transcription kit (Qiagen, Germany). SYBR-Green (Takara, Japan) was used for the RT-qPCR detection. The expression levels were normalized to those of *GAPDH*. The primer sequences used in this step are listed in Supplementary Table S1. The resulting data were analyzed using the 2^−ΔΔCT^ method.

### Statistical analysis

We used Perl software (version 5.18.2) to complete and process the data in this study, and R software (version 4.1.2) was used for the statistical analyses. The independent-sample t-test and Wilcoxon rank-sum test were used to investigate differences between the continuous variables; *p* < 0.05 was typically regarded to be statistically significant.

## Results

### Differential expression analysis

**FIGURE 1:**
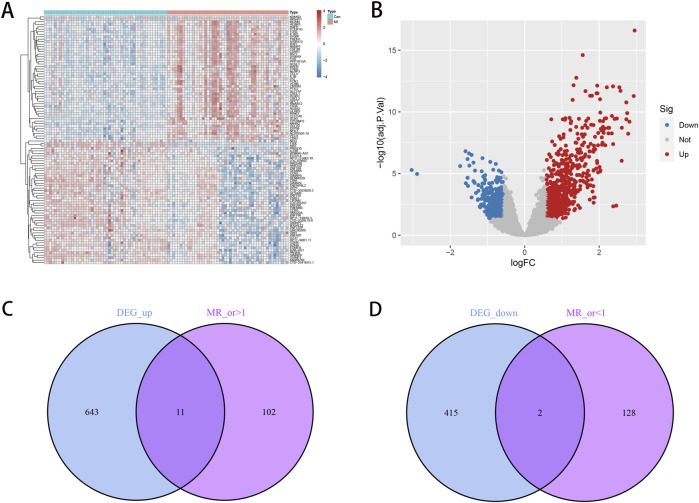
Expression of differential genes and identification of candidate genes. **(A)** Heatmap of the differentially expressed genes (DEGs), where the vertical coordinates represent gene clustering, horizontal coordinates represent sample clustering, color scale represents gene expression abundance, red color represents gene upregulation, and blue color represents gene downregulation. **(B)** Volcano plot of the DEGs, where each dot represents a detected gene; the more off-center the dot, the greater is the diversity of difference; the closer the dot is to the top of the plot, the more significant is the difference. Here, blue dots represent downregulated genes, red dots represent upregulated genes, and gray represents genes that are not differentially expressed. **(C)** Venn diagram of genes with downregulated expression. **(D)** Venn diagram of genes with upregulated expression.

We obtained 1,071 DEGs between healthy individuals and patients with MI. The heatmap in Figure 1A shows the top-50 genes whose expression was significantly increased or decreased. The volcano map in Figure 1B reveals the overall distribution of expression and fold changes of the DEGs.

### MR analysis

We obtained a total of 26,152 SNPs as the instrumental variables, all of which adhered to the three basic assumptions of MR. All selected SNPs had F-statistics exceeding 10 (Supplementary Table S2). Using MR analysis and the three established filtering criteria, we identified 243 MI-related genes (Supplementary Table S3).

**FIGURE 2:**
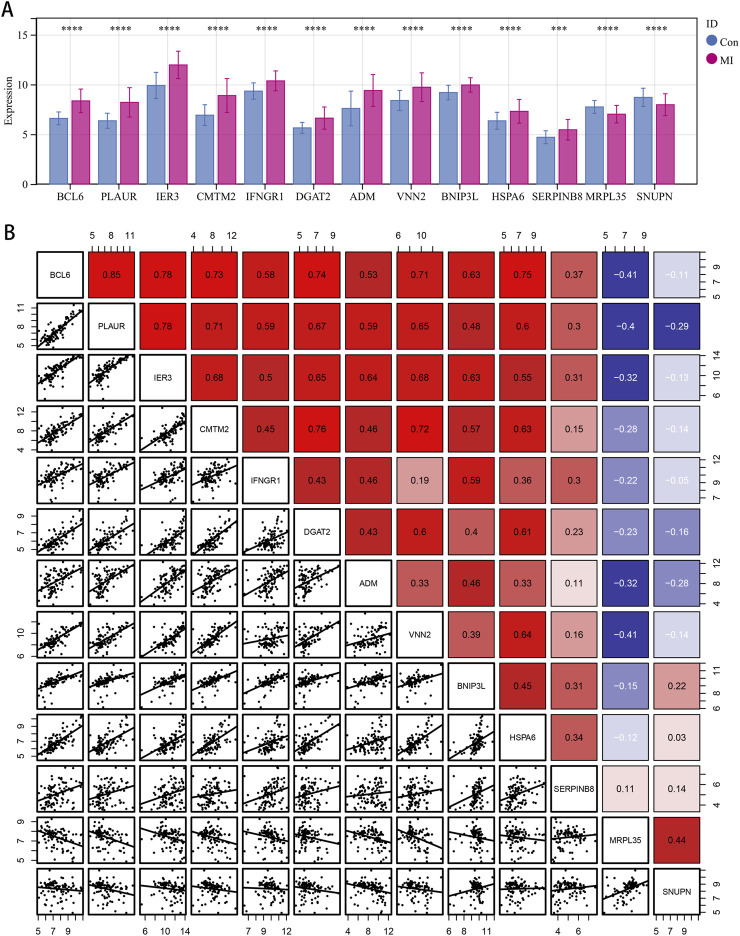
Expression levels of the candidate genes and their correlation analysis. **(A)** Boxplots of the 13 candidate genes identified between the control and myocardial infarction (MI) groups. *p < 0.05, **p < 0.01, ***p < 0.001, and ****p < 0.0001. **(B)** Correlation analysis among the 13 candidate genes.

Through further intersection, we obtained genes that were coexpressed with the disease-related genes and DEGs. We found a total of 13 overlapping genes, where 2 genes *MRPL35* and *SNUPN* showed downregulated expression in MI, and the remaining 11 genes (*ADM*, *BCL6*, *BNIP3L*, *CMTM2*, *DGAT2*, *HSPA6*, *IER3*, *IFNGR1*, *PLAUR*, *SERPINB8*, and *VNN2*) showed upregulated expression (Figures 1C,D). The associations between the independent genetic instrumental variants of the 13 genes in MI are shown in Supplementary Table S4. The expression of these genes is shown in Figure 2A. Based on analysis of the expression levels shown in Figure 2B, we can clearly observe the correlation characteristics among these genes; here, *MRPL35* and *SNUPN* expression was primarily negatively correlated with those of the remaining 11 genes, whereas the expression among the 11 upregulated genes was consistently positively correlated. These observations confirm the crucial biological roles of the identified genes in the pathological process of MI. We performed MR studies on these 13 coexpressed genes to determine the causal contribution of each gene to MI.

**FIGURE 3:**
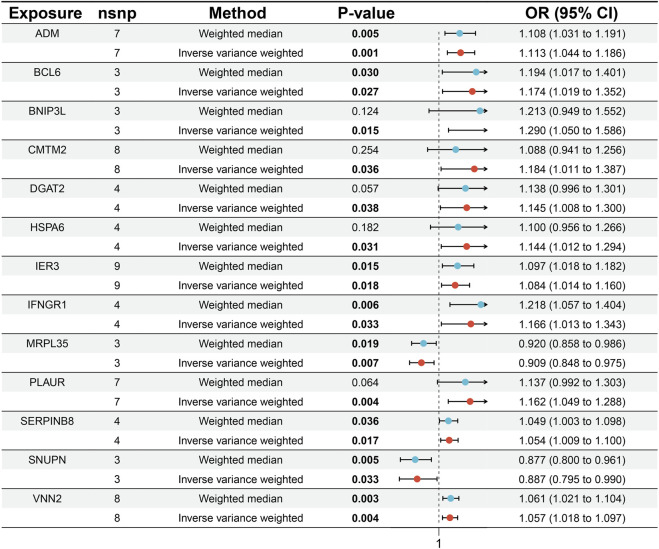
Forest plot of the Mendelian randomization results for the 13 candidate genes and MI.

From the results of the IVW technique (Figure 3), we observe substantial positive causal associations between MI and the 11 upregulated coexpressed genes in the MR analysis as follows: *ADM* (OR: 1.133, 95% confidence interval (CI): 1.104–1.186, *p* = 0.001), *BCL6* (OR: 1.174, 95% CI: 1.019–1.352, *p* = 0.027), *BNIP3L* (OR: 1.290, 95% CI: 1.050–1.586, *p* = 0.015), *CMTM2* (OR: 1.184, 95% CI: 1.011–1.387, *p* = 0.036), *DGAT2* (OR: 1.145, 95% CI: 1.008–1.300, *p* = 0.038), *HSPA6* (OR: 1.114, 95% CI: 1.012–1.294, *p* = 0.031), *IER3* (OR: 1.084, 95% CI: 1.014–1.160, *p* = 0.018), *IFNGR1* (OR: 1.166, 95% CI: 1.013–1.343, *p* = 0.033), *PLAUR* (OR: 1.162, 95% CI: 1.049–1.288, *p* = 0.004), *SERPINB8* (OR: 1.054, 95% CI: 1.009–1.100, *p* = 0.017), and *VNN2* (OR: 1.057, 95% CI: 1.018–1.097, *p* = 0.004). Conversely, both of the downregulated coexpressed genes showed significant negative causal relationships with MI: *MRPL35* (OR: 0.909, 95% CI: 0.848–0.975, *p* = 0.007) and *SNUPN* (OR: 0.887, 95% CI: 0.795–0.990, *p* = 0.033).

For additional validation, we considered the weighted mode, weighted median, and simple mode in addition to MR-Egger. These approaches significantly affected the 13 genes as follows. For the 11 upregulated genes, all approaches showed consistently increased risk of MI (OR > 1). In contrast, all methods consistently showed decreased risk of MI (OR < 1) for the two downregulated genes (Supplementary Table S5).

No statistical significance was detected, and we did not consider it necessary to assess the effects of heterogeneity and pleiotropy because the corresponding tests for the coexpressed genes suggested *p* > 0.05 (Supplementary Table S6). The robustness of the analysis was demonstrated by the sensitivity analysis, which revealed that the effect sizes of the instrumental variants were similar to the total effect size (Supplementary Figure S1).

### GO and KEGG enrichment analyses

**FIGURE 4:**
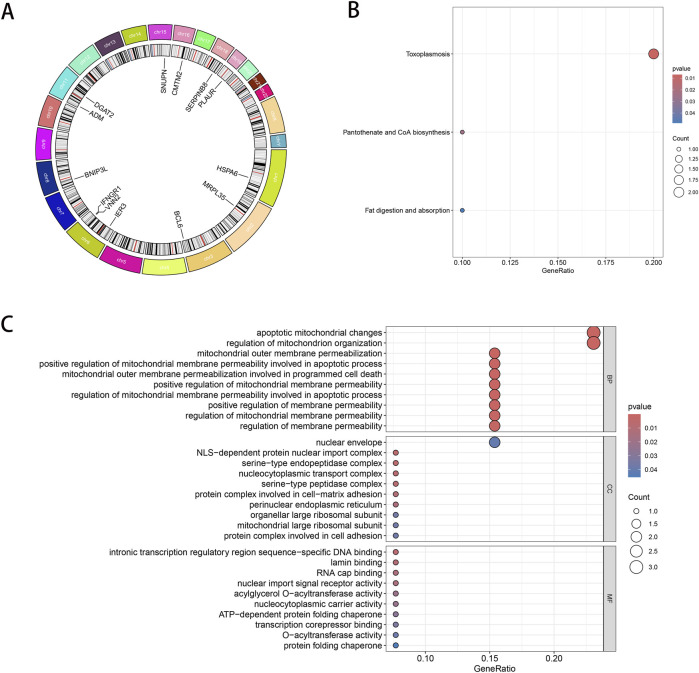
Chromosomal localization and enrichment analysis of the genes. **(A)** Chromosomal distribution of the candidate gene expression (circos circle plot). The first or outer circle is the chromosome where the gene is located and the location information; the second circle shows the copy number changes, with red being an increase in copy number increase and blue being a decrease in copy number; the third circle shows the names of the genes. **(B)** Bar graph demonstrating the KEGG enrichment analysis results of the candidate genes. **(C)** Bar graph showing the GO functional enrichment analysis results of the candidate genes.

The positions of the 13 candidate genes on the chromosomes are displayed in Figure 4A: *MRPL35* and *SNUPN* were found on chromosomes 2 and 15, respectively; *CMTM2* was found on chromosome 16; *PLAUR* was located on chromosome 19; *HSPA6* was located on chromosome 1; *BCL6* was located on chromosome 3; *VNN2*, *IFNGR1*, and *IER3* were located on separate regions of chromosome 6; *BNIP3L* was located on chromosome 8; *DGAT2* and *ADM* were located on chromosome 11. The GO and KEGG pathway enrichment analyses revealed these candidate genes. Multiple terms related to the regulation of membrane permeability associated with GO biological processes were observed to be enriched; these terms included mitochondrial membrane permeability and its regulation for apoptotic processes. In terms of the cellular components, enrichments were observed for the nuclear membrane, protein complexes, cell adhesion, cell-matrix adhesion, apoptosis-associated mitochondrial alterations, and mitochondrial organization. In terms of molecular functions, enrichments were mainly observed for NLS-dependent nuclear protein import complexes, serine-type peptidase complexes, and nucleoplasm transfer complexes (Figure 4C). Furthermore, KEGG analysis revealed that the pathways associated with “fat digestion and absorption,” “pantothenate and CoA biosynthesis,” and “toxoplasmosis” were enriched (Figure 4B).

### GSEA findings

We performed GSEA to investigate the possible roles of the identified genes in differentiating MI from normal samples and to distinguish distinct activation pathways between genes that were expressed effectively and ineffectively (Supplementary Table S7). GSEA revealed that the genes in the high-expression group were strongly enriched in chemokine signaling pathways and cytokine–cytokine receptor interactions; examples of these genes include P*LAUR*, *BCL6*, *BNIP3L*, *IER3*, *IFNGR1*, *HSPA6*, *DGAT2*, and *VNN2*. In addition to the shared gene set described previously, natural-killer-cell-mediated cytotoxicity was found to be enhanced in the group with highly expressed *PLAUR*. The groups with highly expressed *BNIP3L* and *DGAT2* and their gene set also showed enrichment of the NOD-like receptor signaling pathway. Furthermore, the *IFNGR1* high-expression group exhibited enrichment of ribosomes and the B-cell receptor signaling pathway. Similarly, *HSPA6* was highly expressed and enriched in the Toll-like receptor (TLR) signaling pathway. These findings imply that in the context of high expression of various genes, the expression levels of gene sets involved in immune responses (e.g., chemokine signaling pathways and cytokine interactions), pathogen infections (e.g., leishmaniasis infections), and organelle functions (e.g., lysosomes) are enriched.

### Validation of candidate genes involved in MI

**FIGURE 5:**
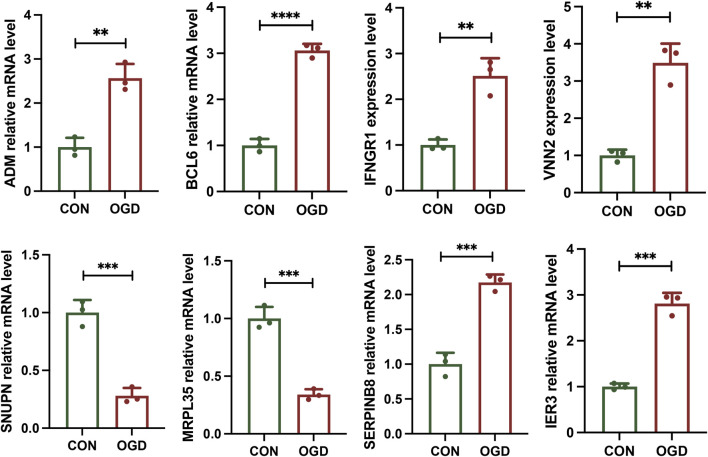
Reverse transcription quantitative polymerase chain reaction results of eight candidate genes. The bar plots represent the relative mRNA expression levels of the following genes in the control (CON) and oxygen–glucose deprivation (OGD) groups: *ADM*, *BCL6*, *IFNGR1*, *VNN2*, *SNUPN*, *MRPL35*, *SERPINB8*, and *IER3*. Among these, the mRNA expression levels of *ADM*, *BCL6*, *IFNGR1*, *VNN2*, *SERPINB8*, and *IER3* were significantly upregulated in the OGD group compared to the CON group, whereas those of *SNUPN* and *MRPL35* were significantly downregulated. The error bars indicate standard error. *p < 0.05, **p < 0.01, ***p < 0.001, and ****p < 0.0001.

To confirm the accuracy of our findings, we selected eight candidate genes for experimental validation based on the following criteria: significant causal associations with MI in both the IVW and weighted median methods (*p* < 0.05 for both methods). We then created a cellular model of MI by subjecting AC16 cells to OGD. The RT-qPCR results demonstrated that compared to the control group, the OGD group had significantly upregulated mRNA expression of *ADM*, *BCL6*, *IFNGR1*, *VNN2*, *SERPINB8*, and *IER3*, whereas *SNUPN* and *MRPL35* had significantly downregulated mRNA expression (all *p* < 0.05), consistent with the MR findings (Figure 5).

## Discussion

The pathogenesis of MI involves genetic susceptibility and mitochondrial dysfunction. In this study, we identified 243 genes responsible for MI based on eQTL and MR analyses; accordingly, thirteen candidate genes were screened by crossing the eQTL results with the mRNA expression profiling results. In addition, enrichment analyses were performed to examine the biological functions of these candidate genes, whose findings demonstrated that the genes were primarily associated with pathways regulating toxoplasmosis, mitochondrial tissue regulatory pathways, and alterations related to apoptosis in the mitochondria. The present study focused on the clinical significance of the intimate physiopathological links among mitochondria, genes, and MI.

Crosstalk between the endothelial cells (ECs) and cardiomyocytes within the cardiac microenvironment is a critical determinant of the myocardial response to ischemic injury. Although ECs and cardiomyocytes originate from distinct lineages, they share stress responses within the cardiac microenvironment. ECs are abundant and metabolically active in the cardiac microvascular bed and serve as “first responders” to stress signals like hypoxia. They also continuously regulate the behaviors and fates of adjacent cardiomyocytes through paracrine mechanisms. Arolkar et al. (2023) reported that the numbers of peripheral ECs increase sharply after acute MI and that their transcriptomes exhibit high expression of proangiogenic genes like *VEGF-A* and *Ang-2*, which drive collateral circulation remodeling; furthermore, they express cardiac-specific contractile proteins (*Tnnt2*, *Myl3*, *Myh6*, etc.) at low but detectable levels. These “cardiomyocyte-like” transcriptional signatures originate from retained epigenetic memory in a subset of cardiac ECs, suggesting that they share a part of the transcriptional regulatory network with cardiomyocyte precursors during embryonic development (Jambusaria et al., 2020; Yucel et al., 2020). In addition, ECs export signals to cardiomyocytes via exosomes to regulate their functions. Hypoxia-preconditioned ECs release extracellular vesicles enriched with miR-126 and miR-210, which can target cardiomyocytes or cardiac progenitor cells to activate the PI3K/Akt and HIF-1α-mediated downstream survival pathways, thereby increasing the ischemic stress tolerance thresholds of both cell types synchronously (Chistiakov et al., 2016). In summary, although ECs and cardiomyocytes differ significantly in terms of lineage and functions, they share overlapping responses to ischemic stress.

Vanins are a family of pantetheinases comprising *VNN1*, *VNN2*, and *VNN3* that have been widely implicated in fatty acid metabolism, oxidative stress, cell migration, and inflammatory responses (Mariani and Roncucci, 2017). Among these, *VNN2* is predominantly expressed in human neutrophils and monocytes, where it is primarily stored within secretory vesicles (Soler et al., 2022). In patients with MI, *VNN2* regulates neutrophil adhesion by physically binding to Mac-1 (CD11b/CD18), thereby promoting neutrophil recruitment to ischemic and hypoxic cardiomyocytes as well as exacerbating inflammation and mitochondrial damage (Yu et al., 2022). Similarly, GSEA revealed that increased *VNN2* expression is associated with activation of the TLR signaling pathway. TLRs constitute an essential class of pattern recognition receptors that primarily activate the nuclear factor-κB (NF-κB) signaling pathway to promote pro-inflammatory cytokine production and cell survival (Dar et al., 2014). Increased *VNN2* expression may activate TLR signaling, resulting in neutrophil accumulation and exacerbation of inflammatory responses in patients with MI.

Mitochondrial functions regulate the survival and activities of neutrophils in hypoxic environments. Recent evidence indicates that the mitochondria not only regulate neutrophil apoptosis and neutrophil extracellular trap formation but also increase neutrophil stability and survival under hypoxic conditions. Under hypoxic conditions, neutrophils maintain the mitochondrial membrane potential and release mitochondrial reactive oxygen species (mROS) by enhancing glycolysis and the 3-phosphate (G-3-P) shuttle mechanism to stabilize the transcription factor HIF-1α and facilitate oxygen delivery to the hypoxic areas (Naffah de Souza et al., 2017). Notably, glycerol-3-phosphate dehydrogenase 2 (GPD2) is a critical mitochondrial enzyme in the G-3-P shuttle pathway that plays a pivotal role in promoting mROS generation, HIF-1α stabilization, and neutrophil survival under hypoxic conditions (Maldarelli and Noto, 2024).

Numerous experiments have shown that adrenomedullin (ADM) plays diverse roles in antimicrobial and inflammatory responses (Xu et al., 2022). In addition to its vasodilatory and hypotensive effects, ADM inhibits NF-κB activation by activating the cAMP/PKA signaling pathway and modulating the transcription of anti-inflammatory cytokines (Schönauer et al., 2017). Simultaneously, ADM induces differentiation of T cells that produce IFN-γ by phosphorylating AKT and STAT3 or stimulating the production of IL-12 through monocyte macrophages (Kong et al., 2020). These findings are also in line with our observations that ADM expression is beneficially associated with monocyte macrophages and improves the prognosis of heart failure after MI (Voors et al., 2019). Long-term ADM injection was shown to delay the transition from left ventricular hypertrophy to heart failure, supporting its potential as both a prognostic biomarker and therapeutic target in patients with MI (Nakamura et al., 2004). The fibrinogen activator urokinase receptor (PLAUR) shows increased expression under hypoxic conditions and is strongly positively correlated with neutrophil abundance (Chen et al., 2021; Dai and Lin, 2023). The mitochondrial ribosomal regulatory subunit MRPL35 is a specific component of the mitochondrial ribosome and is critically involved in the assembly of the cytochrome c oxidase complex (COX) (Box et al., 2017). According to our MR analysis, the upregulation of *MRPL35* expression was associated with reduced risk of MI, suggesting that the cardioprotective role of *MRPL35* is potentially mediated through the attenuation of mROS generation and DNA damage in the cardiomyocytes (Zhang et al., 2019). However, elucidation of the precise molecular mechanisms requires further experimental efforts. *SNUPN* is a protein-coding gene linked to cancer, chronic lymphocytic leukemia, and other conditions (Hao et al., 2023). The functions of *SNUPN* in relation to MI have not been investigated in previous studies. *SNUPN* has been shown to be negatively associated with both resting dendritic cells and active mast cells in the immunological milieu. These findings suggest that *SNUPN*-related pathways may be involved in the induction of immune dysregulation following MI. Further investigations are therefore necessary to fully elucidate the role of *SNUPN* in the context of MI.

*BNIP3L*-mediated mitophagy plays a dual regulatory role in myocardial injury. The corresponding candidate genes are primarily enriched in two pathways, namely, those regulating mitochondrial organization and those related to apoptotic alterations in the mitochondria. Additionally, mitochondrial autophagy is crucial for maintaining mitochondrial quality during MI. *BNIP3L* as a mitochondrial autophagy receptor can bind to Atg8 and eliminate damaged mitochondria through autophagy during cellular development or under pathological conditions (Tong and Sadoshima, 2016). By detecting various intracellular or external stressors, the mitochondria can link the targets with autophagosomes via mitochondrial autophagy receptors for disintegration under stressful circumstances. Hypoxia is a major stressor that triggers *BNIP3L*-mediated mitophagy. These findings highlight *BNIP3L* as a putative target for autophagy in mitochondria subjected to stroke intervention (Wu et al., 2021). Hypoxia-inducible factor 1 (*HIF1A*) stimulates *BNIP3L* under hypoxic conditions caused by interruption of blood flow from coronary artery obstruction due to MI (Li et al., 2021). *HIF1A* initiates LC3-dependent mitochondrial autophagy and promotes the overproduction of mROS. This finding is consistent with the GSEA results, which revealed that the NOD-like receptor signaling pathway was enriched in upregulated *BNIP3L* expression. Additionally, upregulated *BNIP3L* expression is associated with mitochondrial autophagy, which inhibits the activation of NLRP3 inflammatory vesicles in the NOD-like receptor signaling pathway and reduces inflammatory response production. Prior research has shown that *BNIP3L*-induced cardiomyocyte death contributes to cardiac remodeling and indirectly increases fibrosis (Liu et al., 2017). Therefore, regulating *BNIP3L*-mediated mitochondrial autophagy may be a novel therapeutic strategy for improving myocardial injury by reducing inflammation and protecting mitochondrial functions.

Notwithstanding the findings, the present study has several limitations that require further exploration and improvement. First, bidirectional MR was not performed to rule out reverse causality. Second, colocalization analysis was not conducted to confirm whether the identified eQTLs and MI GWAS signals shared the same causal variants, which could affect the robustness of candidate gene inference. Third, when GWAS data are used to explore non-linear associations or stratified effects, there may be challenges from potential heterogeneity owing to differences in sex, age, and health status, which increases the difficulty of analysis. Fourth, as the eQTL summary data were sourced from GWAS databases primarily comprising participants of European ancestry, the present findings are limited to individuals of European descent. Hence, studies including populations with diverse genetic backgrounds are essential in the future to determine the broader applicability of these findings. Fifth, the impacts of post-transcriptional regulation on the phenotypes must be addressed. Therefore, combining multiomics analysis methods like proteomics and metabolomics is necessary to elucidate the regulatory mechanisms more accurately. Sixth, the present study is primarily focused on bioinformatics analysis and *in vitro* cell experiments. Although these methods provide valuable insights, their translational potential could benefit from future studies that directly assess the clinical applicability of the identified genes and pathways in patient cohorts. Finally, despite the differences in study populations and incomplete overlapping between the gene lists tested using eQTL analysis and mRNA expression profiles, we note that we cannot exclude the potential clinical value of the identified genes. For the unique genes identified in both analyses, further research efforts are necessitated to explore the potential biological significance and clinical applications.

## Conclusion

The present study provides some fresh perspectives on the complex pathophysiology of MI. This work entails a large-scale and thorough examination of MI utilizing MR analysis, mRNA expression profiling, and eQTL analysis to reveal the significant associations between 13 candidate genes and MI as well as the mitochondria associated with them. In the future, these genes could become potential targets for medications and research on the molecular mechanisms underlying MI.

## Data Availability

The datasets presented in this study can be found in online repositories. The names of the repositories and accession numbers can be found in the article/Supplementary Material.
