# Supplementary material for: Integrated expression quantitative trait loci and Mendelian randomization analyses of the candidate genes and pathways identified for myocardial infarction
Source: Front Mol Biosci. 2026 Mar 11;13:1693113. doi: 10.3389/fmolb.2026.1693113 (PMC13014038; doi:10.3389/fmolb.2026.1693113)
Supplement: Supplementary file 8 [file Image1.pdf]

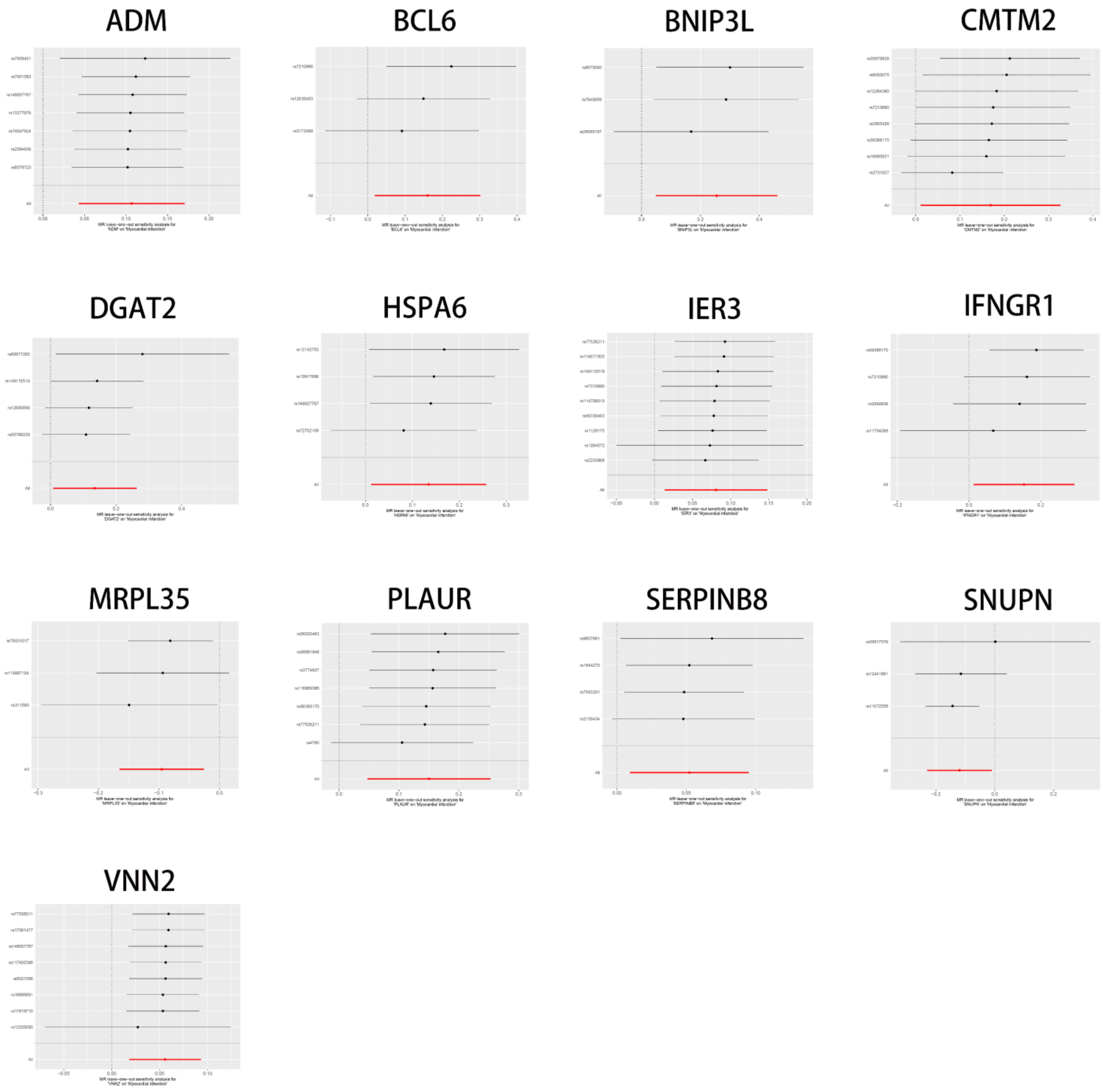

Supplementary Figure S1. MR leave−one−out sensitivity analysis for candidate genes. The x-axis represents the impact of SNPs on exposure and the y-axis represents the impact of SNPs on outcomes. A slope greater than 0 indicates that the exposure factor is an unfavorable factor for the outcome.
